# MiR‐146a Reduces Inflammation in Experimental Pancreatitis via the TRAF6–NF‐κB Signaling Pathway in Mice

**DOI:** 10.1002/iid3.70163

**Published:** 2025-02-28

**Authors:** Xiaoyu Yang, Yuping Ren, Xueyang Li, Liang Xia, Jianhua Wan

**Affiliations:** ^1^ Jiangxi Provincial Key Laboratory of Digestive Diseases, Department of Gastroenterology, Jiangxi Clinical Research Center for Gastroenterology, Digestive Disease Hospital, The First Affiliated Hospital, Jiangxi Medical College Nanchang University Nanchang Jiangxi China; ^2^ Department of Rheumatology, The First Affiliated Hospital, Jiangxi Medical College Nanchang University Nanchang Jiangxi China

**Keywords:** acute pancreatitis, inflammatory, miR‐146a, NF‐κB, TRAF6

## Abstract

**Background:**

The initial inflammatory response plays a pivotal role in the development of acute pancreatitis. MiR‐146a is believed to play a key role in negatively regulating inflammation and potentially contributes to anti‐inflammatory activity in acute pancreatitis, though its mechanism remains largely unexplored.

**Objectives:**

This study aimed to explore the effects of miR‐146a on AP in mice and clarify its regulatory mechanisms in pancreatic inflammation and damage.

**Methods:**

Adult male BALB/C mice were used. Adeno‐associated virus (AAV) vectors were used to modulate miR‐146a expression in mice via tail vein injection. AP was induced by intraperitoneal injection of caerulein, caerulein + LPS, or l‐arginine. Histological analysis, immunohistochemistry staining, immunofluorescence staining, measurements of amylase and lipase activities, and qRT‐PCR were performed.

**Results:**

Overexpression of miR‐146a reduced pancreatic damage and inflammation in caerulein‐induced AP. It decreased serum amylase and lipase levels, mitigated pathological features such as interstitial edema and inflammatory cell infiltration in the pancreas and lung, and reduced neutrophil infiltration and proinflammatory cytokine expression. MiR‐146a attenuated the activation of the NF‐κB signaling pathway by inhibiting the degradation of IκBα and the expression of phosphorylated‐p65 and reducing the nuclear translocation of NF‐κB p65. Similar protective effects of miR‐146a were observed in AP models induced by l‐arginine and caerulein combined with LPS.

**Conclusions:**

MiR‐146a alleviates acute pancreatitis in mice by targeting TRAF6 and suppressing the activation of the NF‐κB signaling pathway. These findings suggest that miR‐146a could be a potential therapeutic target for AP.

## Graphical abstract

Overexpression of miR‐146a leads to reduced pancreatic damage and inflammation by downregulating the TRAF6/NF‐κB pathway. By examining different models of AP (induced by caerulein, l‐arginine, and caerulein combined with LPS), the research underscores miR‐146a's broad applicability in reducing pancreatic inflammation and damage across various AP contexts. MiR‐146a as a promising target for therapeutic intervention in AP, given its significant role in dampening inflammation through the TRAF6/NF‐κB pathway.

**Abbreviations**
AAV: adeno‐associated virus
AP: acute pancreatitis
IP: intraperitoneal
miR‐146a sponge + Cn: caerulein + miR‐146a sponge treatment
miR‐146a + Cn: caerulein + miR‐146a overexpression treatment
NF‐κB: nuclear factor κB
PBS+Cn: caerulein treatment
PBS + NS: normal saline treatment
TRAF6: tumor necrosis factor receptor‐associated factor 6

## 1 Introduction

Acute pancreatitis (AP) is an acute inflammatory condition of the pancreas, initiated by the abnormal activation of digestive enzymes. The incidence of AP has been rising annually, a trend possibly linked to environmental, dietary, genetic, and other factors [1]. Although great progress has been made in understanding the pathophysiological mechanism of AP and developing clinical therapeutic methods for it, the mortality rate among AP patients remains high [2], mainly due to systemic inflammatory response syndrome (SIRS) and multiorgan failure in the early stage [3]. Therefore, effectively mitigating inflammation to decrease the incidence of SIRS and organ failure represents a crucial early treatment strategy [4]. Consequently, a deeper insight into the inflammatory mechanisms in AP could pave the way for more effective therapeutic interventions, thereby enhancing disease prognosis.

Nuclear factor κB (NF‐κB), a transcriptional regulator implicated in inflammation, immune responses, and cancer biology, belongs to a family comprising three types of proteins: IKK proteins, IκB proteins, and NF‐κB proteins [5]. Its activation typically involves the ubiquitin‐dependent degradation of IκBα and the subsequent nuclear translocation of NF‐κB dimers, leading to the release of several proinflammatory cytokines, including TNF‐α, IL‐1, IL‐6, and IL‐8. Experimental studies in pancreatitis have identified NF‐κB pathway activation as an early marker in the progression of AP. With its activation in acinar cells exacerbating the systemic inflammatory response and the severity of pancreatitis in mice [6, 7]. The deletion of the specific pancreatic NF‐κB p50 precursor protein has been shown to mitigate the severity of caerulein‐induced pancreatitis and reduce oxidative stress [8]. Consequently, targeting NF‐κB activation represents a critical strategy for mitigating AP [9, 10]. MicroRNAs (miRNAs), short single‐stranded RNAs of about 22 nucleotides long, play a crucial role in modulating inflammatory signals and have been increasingly recognized for their significance [11, 12]. MiR‐146 was initially identified as an immune modulator affecting microbial infections in mammals. MiR‐146a predicts an increased in‐hospital death risk in both severe acute pancreatitis (SAP) and moderately severe acute pancreatitis (MSAP) patients and exhibits potential as a marker for AP management and prognosis [13]. Subsequent studies have elucidated miR‐146a's role as a primary inhibitor of NF‐κB activation, specifically by targeting IRAK1/TRAF6 within the MyD88‐dependent pathway [14], and suppressing NF‐κB‐mediated inflammation in vivo [15]. Recent findings further highlight miR‐146a's critical regulatory role in the formation of neutrophil extracellular traps (NETs) [16], NETs' early‐stage deposition in pancreatic tissue, and their contribution to AP exacerbation [17]. Thus, miR‐146a plays an integral role in connecting inflammation with AP. Nonetheless, the specific regulatory mechanisms of miR‐146a in AP inflammation remain to be fully understood. This study investigated the effects of miR‐146a on the course of AP by regulating the TRAF6/NF‐κB signaling pathway.

## 2 Materials and Methods

### 2.1 Mice Treatments and Reagents

All the experimental procedures involving animals were given the green light by the Animal Care Committee of the Institutional Animal Care and Use Committee at The First Affiliated Hospital of Nanchang University (with the Ethical Approval Number 2017024), and the entire process was carried out in strict compliance with the guidelines set by the said committee. Adult male BALB/C mice (6–8 weeks old) purchased from Hunan SJA Laboratory Animal Co. Ltd. (HSLAC, Hunan, China) were maintained at 20°C–25°C, with 50%–70% humidity and a 12 h light cycle. Food and water were available ad libitum. AP was generated by a series of 10 intraperitoneal (IP) injections of caerulein (50 μg/kg body weight/h); the caerulein plus LPS model was induced by IP injection of lipopolysaccharide (5 mg/kg) immediately after the caerulein injections [18]. Two IP injections of l‐arginine solution (8%, pH = 7.4) with a dose of 4 g/kg/h were used to construct the l‐arginine pancreatitis model. Caerulein (catalog mo. C9026), LPS (catalog no. L4130), and l‐arginine (catalog no. A5006) were purchased from Sigma‐Aldrich (St. Louis, MO, USA). Mice were euthanized 24 h after the last caerulein injection and 72 h after the last l‐arginine injection. Control mice were given saline solution injections. During the experiment, the researchers strictly followed the National Institutes of Health's “Principles for the Care of Laboratory Animals” (revised in 1996) and the National Science and Technology Commission of China's “Regulations on Animal Management” (revised in 2017) to minimize animal suffering and reduce the number of animals used in the experiment. At the end of the experiment, all the animals were killed in a scientifically standardized manner.

### 2.2 Construction of and Infection With Recombinant AAV‐miR‐146a and AAV‐miR‐146a Sponge

The pAAV9‐U6‐GFP (adeno‐associated virus (AAV)) vectors carrying miR‐146a‐5p (MIMAT0000158; GATCCGTGAGAACTGAATTCCATGGGTTTCAAGAGAACCCATGGAATTCAGTTCTCATTTTTTA), miR‐146a‐5p sponge (MIMAT0000158; CGCAACCCATGGAAATAGTTCTCAGGGTCCCAACCCATGGAAATAGTTCTCAGGGTCCCAACCCATGGAAATAGTTCTCAGGGTCCCAACCCATGGAAATAGTTCTCAA) or a negative control were generated (Vigene Bioscience, Jinan, China). AAV‐miR‐146a and AAV‐miR‐146a sponge were injected via the tail vein with 50 μL PBS per mouse [19]. The mice showed adequate transfection 3 weeks after AAV‐9 (1 × 10^12^) administration.

### 2.3 Histological Analysis

Then, pancreas and lung tissues were fixed in 4% paraformaldehyde for 24 h, followed by embedding in paraffin. The 3‐μm sections were deparaffinized and stained with hematoxylin and eosin (H&E). Histological scoring was performed independently and blind by two pathologists scored for edema, inflammatory cell infiltration, hemorrhage, fat necrosis, and acinar necrosis as previously described [20].

### 2.4 Immunohistochemistry Staining

For immunohistochemistry staining, the sections were incubated overnight at 4°C with primary antibodies against myeloperoxidase (anti‐MPO (ab134132, Abcam), anti‐IκBα (ab32518, Abcam), anti‐phospho‐p65 (phosphor S536) (ab86299, Abcam)) after blocking endogenous peroxidase with 3% H_2_O_2_. After washing, the sections were incubated with secondary antibodies, followed by addition of a 3,3‐diaminobenzidine (DAB) solution for development and counterstaining with hematoxylin. The staining intensity of each section was observed by light microscopy (CKX41, Olympus, Tokyo, Japan) at a magnification of 200×. Two experienced pathologists evaluated the samples independently and blind based on positive staining and staining intensity and then scored them separately as follows: 0 (0%), 1 (1%–25%), 2 (26%–50%), 3 (51%–75%), and 4 (76%–100%) for positive staining; 0 (normal), 1 (weak), 2 (medium), and 3 (strong) for staining intensity [21].

### 2.5 Immunofluorescence Staining

The sections were fixed with 4% paraformaldehyde for 1 h and permeabilized in 0.1% Triton X‐100 for 30 min. After blocking in 10% goat serum, the sections were incubated overnight at 4°C with primary antibodies against GFP (catalog no. 6556, Abcam), anti‐NF‐κB p65 (ab16502, Abcam), TRAF6 (ab40675, Abcam), and P‐IKKα/β (ab194845, Abcam) and then with secondary antibodies (Invitrogen) for 1 h in the dark at room temperature. The nuclear fluorochrome 4′,6‐diamidino‐2‐phenylindole (DAPI; 1:5000 dilution, Invitrogen) was used to stain the cell nuclei. The slides were photographed with a fluorescence microscope (Olympus, Tokyo, Japan).

### 2.6 Measurements of Amylase and Lipase Activities

Blood was collected and centrifuged at 3000 *g* for 10 min. After collecting the serum, amylase and lipase activity (U/L) was measured according to the manufacturer's instructions (Jiancheng Biotech, Nanjing, China).

### 2.7 qRT‐PCR

Total RNA, including miRNA and mRNA, was isolated from tissues in the pancreas using a miRNA and mRNA kit (Tiangen, China) according to the manufacturer's instructions. Approximate RNA quantity and quality were estimated using a NanoDrop 2000 Spectrophotometer (Thermo Scientific, Waltham, MA, USA). Reverse transcription was performed with a reaction mixture, and quantitative real‐time polymerase chain reaction (PCR) was performed with a miRNA and mRNA qPCR kit (Tiangen, China) and SYBR Green qPCR Master Mix (Tiangen, China) by using the StepOnePlus real‐time PCR system (ABI, CA, USA). The primers are shown in Table 1. Primers for miR‐146a were obtained from Tiangen. 5S and β‐actin served as the miRNA and mRNA endogenous controls, respectively. The fold change in RNA expression was shown by the 2^−ΔΔCt^ value, which represents the change in expression as measured by the comparative threshold cycle (Ct) method.

**Table 1:** The primer sequences used.

Genes	Primer
TNF‐α	Forward: 5′‐ GACGTGGAACTGGCAGAAGAG ‐3′
	Reverse: 5′‐ TTGGTGGTTTGTGAGTGTGAG ‐3′
IL‐1β	Forward: 5′‐ GCCACCTTTTGACAGTGATGAG ‐3′
	Reverse: 5′‐ AAGGTCCACGGGAAAGACAC ‐3′
IL‐6	Forward: 5′‐ TAGTCCTTCCTACCCCAATTTCC ‐3′
	Reverse: 5′‐ TTGGTCCTTAGCCACTCCTTC ‐3′
GAPDH	Forward: 5′‐ TGATGACATCAAGAAGGTGGTGAAG ‐3′
	Reverse: 5′‐ TCCTTGGAGGCCATGTAGGCCAT ‐3′

### 2.8 Statistical Analysis

The data are presented as the mean ± SEM. Statistical analyses, such as the Mann–Whitney nonparametric *U* test and the two‐tailed Student's *t* test, were carried out with the application of SPSS statistical software version 20.0 (IBM Corp., Armonk, NY, USA). In every case, a *p* < 0.05 was regarded as statistically significant.

## 3 Results

### 3.1 Overexpression of miR‐146a Reduces Pancreatic Damage in Caerulein‐Induced AP

**Figure 1:**
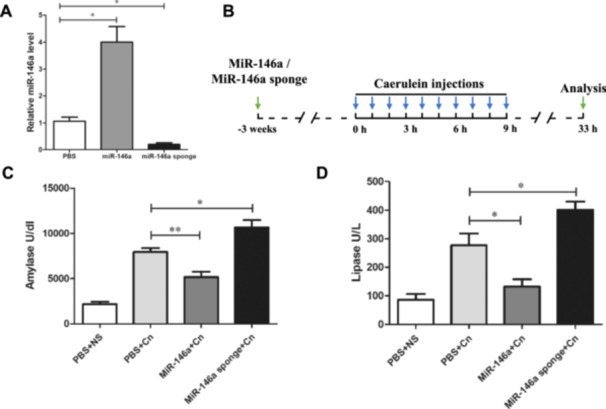
Effects of miR‐146a on enzymatic changes in AP mice. (A) The expression of miR‐146a after treatment with AAV. (B) Design of the mouse experiment (PBS + NS group (*n* = 8), PBS + Cn group (*n* = 8), miR‐146a + Cn group (*n* = 8), miR‐146a‐sponge + Cn (group *n* = 8); caerulein, 50 μg/kg, 10 intraperitoneal injections). (C) Serum amylase and (D) lipase activities are shown. PBS + NS: PBS and saline treatment. PBS + Cn: PBS and caerulein treatment. MiR‐146a + Cn: miR‐146a overexpression and caerulein treatment. MiR‐146a‐sponge + Cn: miR‐146a‐sponge and caerulein treatment. Data shown are the means ± SEM. *p < 0.05, **p < 0.01.

**Figure 2:**
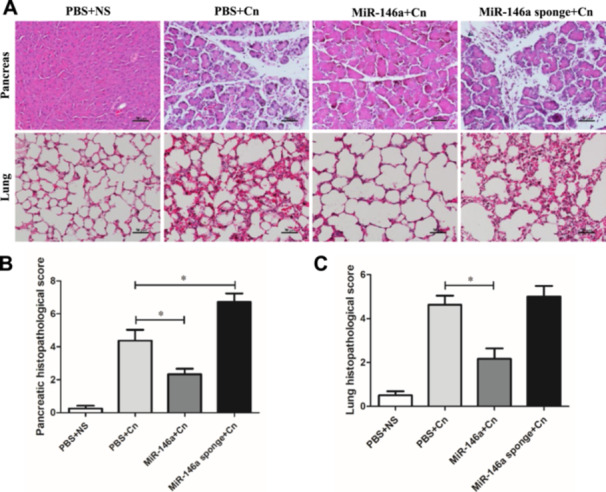
Pathological changes in the pancreas and lungs in AP (*n* = 8 for each group). (A) Pancreas and lung histology (×200). (B) Pancreas pathology scores and (C) lung histology scores in mice are shown. PBS + NS: saline treatment. PBS + Cn: caerulein treatment. MiR‐146a + Cn: miR‐146a overexpression and caerulein treatment. MiR‐146a‐sponge + Cn: miR‐146a‐sponge and caerulein treatment. Data shown are the means ± SEM. *p < 0.05.

To investigate the effect of miR‐146a on experimental AP, mice were administered exogenous AAV‐miR‐146a and AAV‐miR‐146a sponge via tail vein injection. Three weeks later, RT‐PCR analysis confirmed the alteration in miR‐146a expression levels. miR‐146a was significantly elevated in the treated group, while the miR‐146a sponge group showed a reduction compared to the PBS control group (Figure 1A). The expression of the GFP protein in pancreatic acinar cells also indicated successful transfection (Supporting Information S1: Figure 1A). The well‐established AP model as described in previous studies was used. The mice were induced by 10 injections of caerulein 3 weeks after injection with AVV‐miR‐146a or AAVmiR‐146a sponge and sacrificed 24 h after the last injection (Figure 1B). As shown in Figure 1C,D, the levels of serum amylase and lipase decreased with the overexpression of miR‐146a; however, the reduction in miR‐146a levels led to higher levels of serum amylase and lipase compared to the untreated caerulein‐exposed mice. HE staining of the pancreatic tissue in the PBS or AAV‐negative groups treated with normal saline showed a normal pancreas, while the typical pathologic features of AP existed in the PBS or AAV‐negative groups treated with caerulein (Figure 2A and Supporting Information S2: Figure 2A). Histological examination showed that miR‐146a overexpression mitigated the typical pathological features of AP, such as interstitial edema, inflammatory cell infiltration, and pancreatic necrosis, unlike the PBS and caerulein treatment group (Figure 2A,B). The lung histology of AP mice showed highly congested pulmonary interstitials, widened alveolar spaces, and partial atrophy of the pulmonary vesicles [22]. The protective effects of miR‐146a decreased lung damage compared with that in the AP with PBS or miR‐146a sponge groups (Figure 2A,C). Overall, our data suggest that elevated miR‐146a may alleviate the severity of caerulein‐induced AP.

### 3.2 Anti‐Inflammatory Effect of miR‐146a in Caerulein‐Induced AP

**Figure 3:**
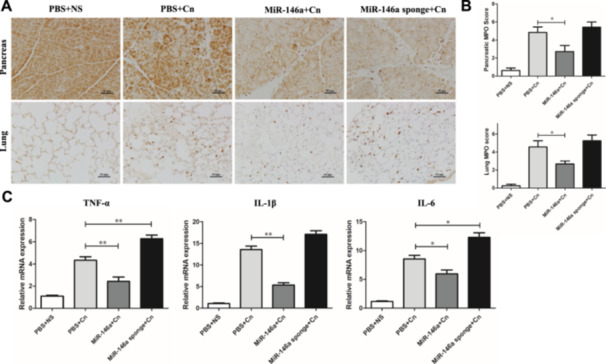
MiR‐146a affects inflammation in AP (*n* = 8 for each group). (A) The expression of MPO in pancreatic and lung tissue (×200). (B) The MPO immunohistochemistry score in pancreatic and lung tissue. (C) The expression of inflammatory cytokines (TNF‐α, IL‐1β, and IL‐6) in pancreatic tissue. PBS + NS: saline treatment. PBS + Cn: caerulein treatment. MiR‐146a + Cn: miR‐146a overexpression and caerulein treatment. MiR‐146a‐sponge + Cn: miR‐146a‐sponge and caerulein treatment. Data shown are the means ± SEM. *p < 0.05, **p < 0.01.

To explore miR‐146a's role in modulating inflammatory responses in AP models, we utilized MPO staining to gauge neutrophil infiltration and proinflammatory cytokine levels. Notably, the AAV‐miR‐146a sponge‐treated and caerulein‐exposed groups without treatment exhibited significantly more MPO‐positive cells in both pancreatic and lung tissues than those treated with miR‐146a (Figure 3A,B). Q‐PCR analysis of inflammatory markers such as TNF‐α, IL‐1β, and IL‐6 revealed that miR‐146a effectively reduced pancreatic inflammation compared to the control AP group, while inflammation markedly increased in the sponge‐treated group (Figure 3C).

### 3.3 MiR‐146a Attenuates the Activation of the NF‐κB Signaling Pathway by TRAF6 in Caerulein‐Induced AP

**Figure 4:**
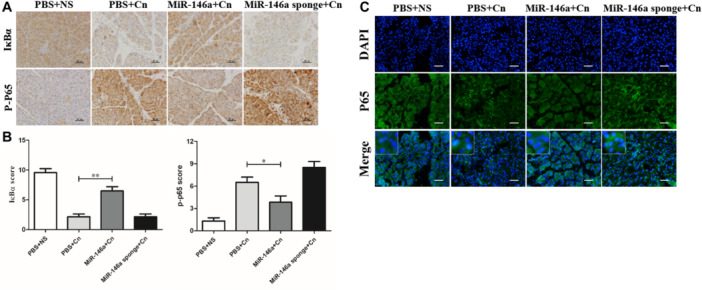
MiR‐146a regulates inflammation via the NF‐κB signaling pathway in AP. (A) The expression of P‐P65 and IκBα (×200) and (B) score in the pancreas are shown. (C) Immunofluorescence staining analysis of P65 in the pancreas is shown (×200). PBS + NS: saline treatment. PBS + Cn: caerulein treatment. MiR‐146a + Cn: miR‐146a overexpression and caerulein treatment. MiR‐146a‐sponge + Cn: miR‐146a‐sponge and caerulein treatment. Data shown are the means ± SEM. *p < 0.05, **p < 0.01.

**Figure 5:**
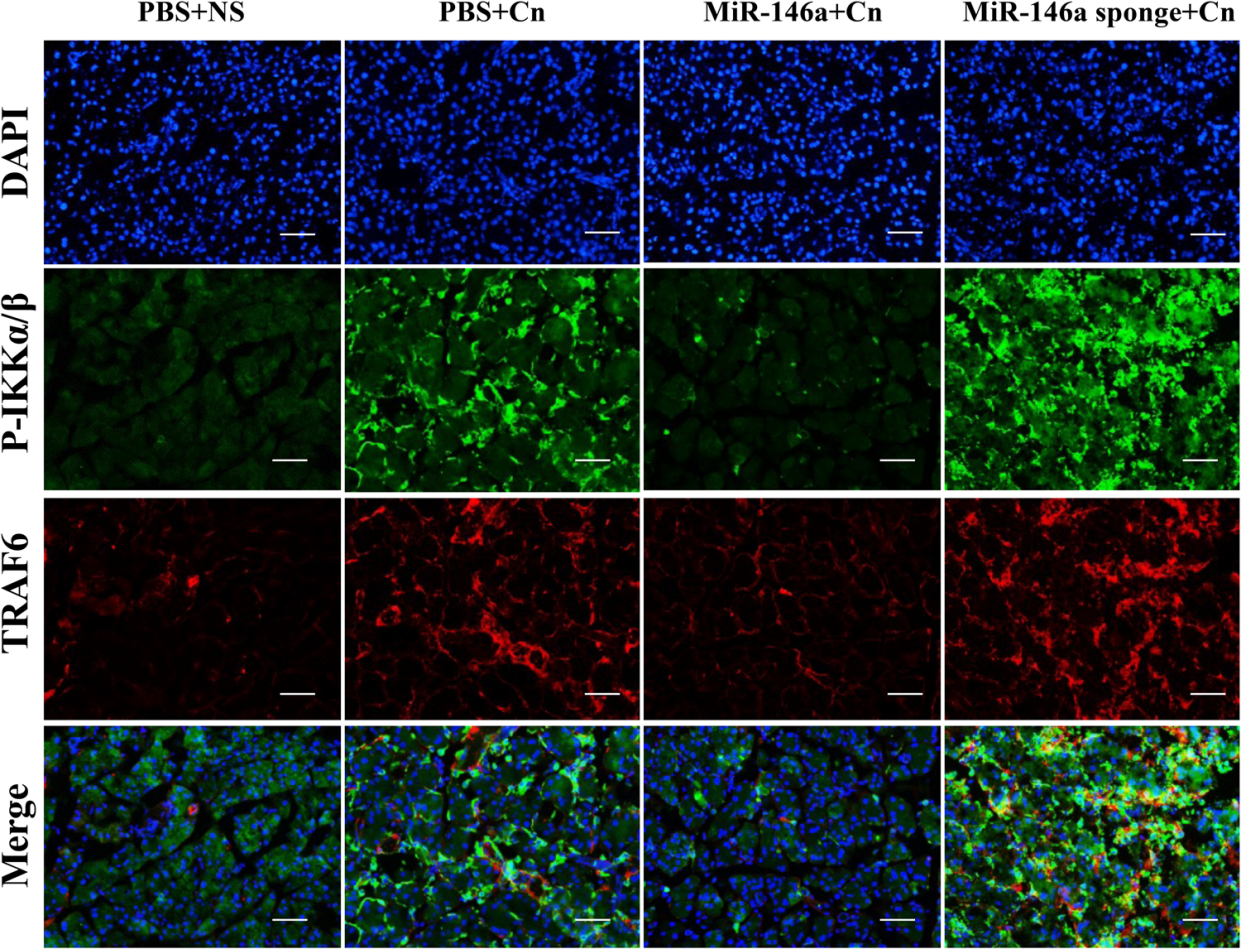
The mechanism of miRNA‐146a regulation in AP via the TRAF6–NF‐κB signaling pathway. Immunofluorescence staining analysis of TRAF6 and P‐IKKα/β in the pancreas is shown (×200). PBS + NS: saline treatment. PBS + Cn: caerulein treatment. MiR‐146a + Cn: miR‐146a overexpression and caerulein treatment. MiR‐146a‐sponge + Cn: miR‐146a‐sponge and caerulein treatment.

The transcription factor NF‐κB serves as a pivotal pathway to inflammatory responses and induces the expression of various proinflammatory mediators [23]. The degradation of IκBα and increased phosphorylated‐p65 expression were significantly observed in caerulein‐induced pancreatitis, which indicated the activation of NF‐κB signaling. IHC staining indicated that miR‐146a inhibited the degradation of IκBα and the expression of phosphorylated‐p65 (Figure 4A,B). The immunofluorescence staining of P65 demonstrated NF‐κB P65 localization, which showed that positive nuclear expression of NF‐κB P65 in cells increased in AP. However, miR‐146a reduced the transfer of the P65 protein from the cytoplasm to the nucleus to reduce the downstream transcription of many inflammation‐related genes (Figure 4C). Based on the miRNA database and previous reports, we assessed TRAF6 and P‐IKKα/β expression by immunofluorescence colocalization and found a direct regulatory relationship between TRAF6 and P‐IKKα/β (Figure 5). Overall, our data suggest that miR‐146a regulates the inflammation of experimental pancreatitis by inactivating transcription factors and the TRAF6–NF‐κB signaling pathway, which attenuates the release of proinflammatory cytokines.

### 3.4 MiR‐146a Reduces Pancreatic Damage in AP Induced by l‐Arginine and Caerulein Combined With LPS

**Figure 6:**
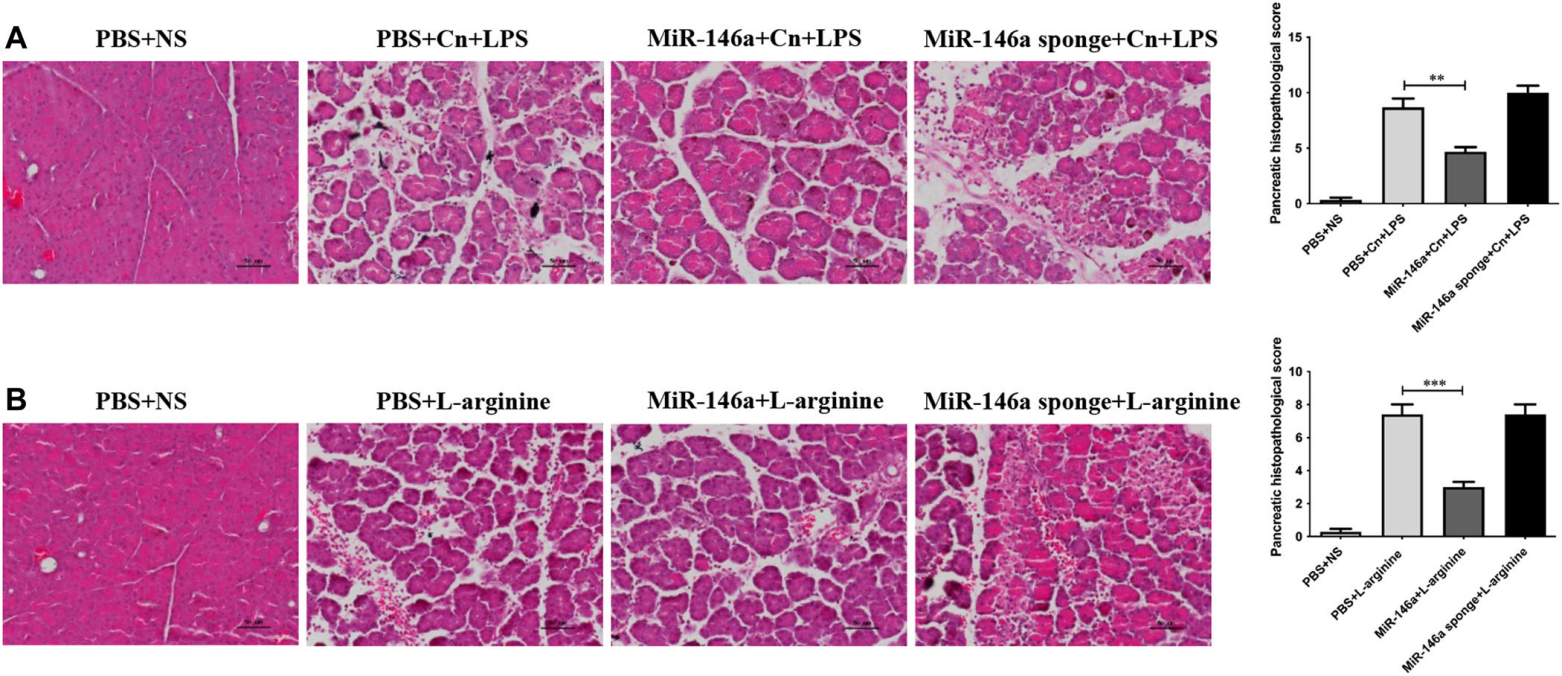
MiR‐146a affects AP in different animal models. (A) Pancreas histology in AP mice mediated by caerulein combined with LPS and (B) l‐arginine is shown (×200). PBS + NS: saline treatment. PBS + Cn + LPS: caerulein and LPS treatment. MiR‐146a + Cn + LPS: miR‐146a overexpression and caerulein and LPS treatment. MiR‐146a‐sponge + Cn + LPS: miR‐146a‐sponge and caerulein and LPS treatment. PBS + l‐arginine: l‐arginine treatment. MiR‐146a + l‐arginine: miR‐146a overexpression and l‐arginine treatment. MiR‐146a‐sponge + l‐arginine: miR‐146a‐sponge and l‐arginine treatment. Data shown are the means ± SEM. **p < 0.01, ***p < 0.001.

Our data suggested that elevated miR‐146a may alleviate the severity of caerulein‐induced AP. To further examine the effect of miR‐146a on experimental AP, we introduced two other animal models of AP (induction with arginine or caerulein in combination with LPS). Although the pathogenesis and severity of AP in these two models are different from those constructed with caerulein alone, the results remained similar. The pancreatic histology of AP mice showed highly edematous acinar cells, widened spaces, and partial necrosis of the pancreas. The protective effects of miR‐146a decreased pancreatic damage, and the miR‐146a sponge aggravated the pathologic features of AP (Figure 6A,B).

## 4 Discussion

Some evidence indicates that managing the inflammatory response in patients can effectively address AP and decrease mortality rates [24, 25]. MiR‐146a is a potent negative regulator of the inflammatory response in a variety of inflammation‐involved disorders [26, 27]. This study highlights miR‐146a's role in suppressing the inflammatory response, showing that its overexpression mitigates pancreatic damage in AP. Notably, our findings underscore miR‐146a's critical role in deactivating the NF‐κB signaling pathway through TRAF6 in AP models. These insights constitute a significant leap forward in understanding miR‐146a's association with AP, underscoring the pivotal influence of the miR‐146a/TRAF6/NF‐κB pathway in the development of pancreatic inflammation and damage.

To determine the anti‐inflammatory role of miR‐146a in AP, we utilized AAV for targeted miR‐146a expression in pancreatic tissue. Recognized for its efficacy in gene transfer [28], AAV has shown exceptional capability for genetic interventions in pancreatic tissue [20]. Following the successful modulation of miR‐146a expression, we established a caerulein‐induced AP model to assess the condition's severity through serum enzymatic levels and pancreatic damage. MiR‐146a overexpression greatly alleviated AP in mice. Furthermore, pathological changes in the lungs, the most common organ involved in AP, were further examined [3, 29]. Our findings were further supported by results from other AP models induced by l‐arginine and a combination of caerulein and LPS [18], suggesting miR‐146a's potential as a therapeutic target for AP.

NF‐κB transcription factors are crucial in numerous biological processes, particularly in host immunity. Recent research has established that unregulated NF‐κB activity leads to inflammation‐related diseases, positioning NF‐κB as a potential target for therapeutic intervention in such conditions [30]. The activation of NF‐κB is a key inflammatory pathway in the development of AP [8], as supported by multiple studies [31, 32]. In AP, ubiquitin‐dependent degradation of IκBα and translocation of NF‐κB dimers to the nucleus are conspicuous, and the expression of interleukin IL‐1β, IL‐6, and TNF‐α as intracellular signals is upregulated by activating NF‐κB [8, 33]. Consistent with extensive research, our findings indicate a reduction of IκBα in pancreatic tissue in the AP model, accompanied by significant accumulation of the key molecule p‐p65, a marker of NF‐κB activation. This process leads to the enhanced expression of inflammatory cytokines (IL‐1β, IL‐6, and TNF‐α) due to NF‐κB activation.

The NF‐κB signaling pathway has been a subject of extensive research due to its crucial role in immune‐related diseases [34, 35]. Significant advancements have been made in elucidating the regulatory signals governing NF‐κB activation [36]. Previous studies have firmly established a close relationship between miR‐146a and NF‐κB [37]. Elevated levels of miR‐146a in monocytes and macrophages have demonstrated the ability to suppress NF‐κB‐driven inflammation [38]. Utilizing miR‐146a mimics targeted specifically at myeloid cells holds promise as a therapeutic approach for inflammatory and myeloproliferative disorders [15]. Notably, the loss of miR‐146a disrupts inflammatory signaling pathways and heightens sensitivity to inflammation [39].

MiR‐146a demonstrated a significant reduction in the degradation of IκBα and the expression of phosphorylated‐p65 in AP mice, suggesting its potential to inhibit the NF‐κB axis‐mediated immune response. Furthermore, TRAF6, a pivotal activator within the NF‐κB signaling pathway, is known to interact with NF‐κB‐inducing kinase [40], a target of miR‐146a as established in previous research [41]. Our findings confirm that TRAF6 directly engages with IKKA/B, thereby modulating the NF‐κB signaling pathway in a mouse model of AP. Drawing from the miRNA database and existing literature, we infer that miR‐146a may exert influence on NF‐κB signaling activation by regulating TRAF6, thereby impacting the pancreatic inflammatory response.

Collectively, our findings align with previous research and underscore the pivotal role of miR‐146a in AP. Effective miR‐146a function inhibited TRAF6 expression and prevented the nuclear translocation of NF‐κB p65, leading to a reduction in proinflammatory cytokines and dampening of the pancreatic immune response. MiR‐146a exerted its anti‐inflammatory effect by modulating the TRAF6/NF‐κB signaling pathway in AP mice. To elucidate the precise molecular mechanisms governing miR‐146a's anti‐inflammatory actions in AP, future studies should consider the manipulation of miR‐146a expression levels in the pancreas, as well as the use of specific antagonists and agonists targeting the TRAF6/NF‐κB signaling pathway or the utilization of knockout transgenic mice.

## 5 Conclusion

In our current study, we have identified miR‐146a as a regulator of anti‐inflammatory mechanisms that alleviate AP in mice. MiR‐146a exerts its negative regulation by targeting TRAF6, and its treatment effectively suppresses the activation of the NF‐κB signaling pathway. Conversely, when miR‐146a is silenced, it promotes NF‐κB signaling activation and exacerbates pancreatic inflammation during AP. The inhibition of the TRAF6/NF‐κB signaling pathway likely accounts for the anti‐inflammatory effects of miR‐146a in the context of AP. These findings suggest that harnessing miR‐146a's negative regulation could hold promise in mitigating the immune response and reducing the severity of AP.

## Author Contributions

**Xiaoyu Yang:** data curation, formal analysis, methodology, project administration, writing – original draft, writing – review and editing. **Yuping Ren:** data curation, formal analysis, methodology, project administration. **Xueyang Li:** methodology, project administration. **Liang Xia:** conceptualization, data curation, formal analysis, funding acquisition, methodology, project administration, supervision, writing – review and editing. **Jianhua Wan:** conceptualization, data curation, formal analysis, funding acquisition, investigation, methodology, project administration, supervision, writing – review and editing. All authors contributed to the manuscript revision, read and approved the submitted version.

## Conflicts of Interest

The authors declare no conflicts of interest.

## Supporting information

Supporting information.

Supporting information.

Supporting information.

## Data Availability

The authors have nothing to report.
