# Supplementary figures and images for: MiR‐146a Reduces Inflammation in Experimental Pancreatitis via the TRAF6–NF‐κB Signaling Pathway in Mice
Source: Immun Inflamm Dis. 2025 Feb 28;13(3):e70163. doi: 10.1002/iid3.70163 (PMC11868994; doi:10.1002/iid3.70163)

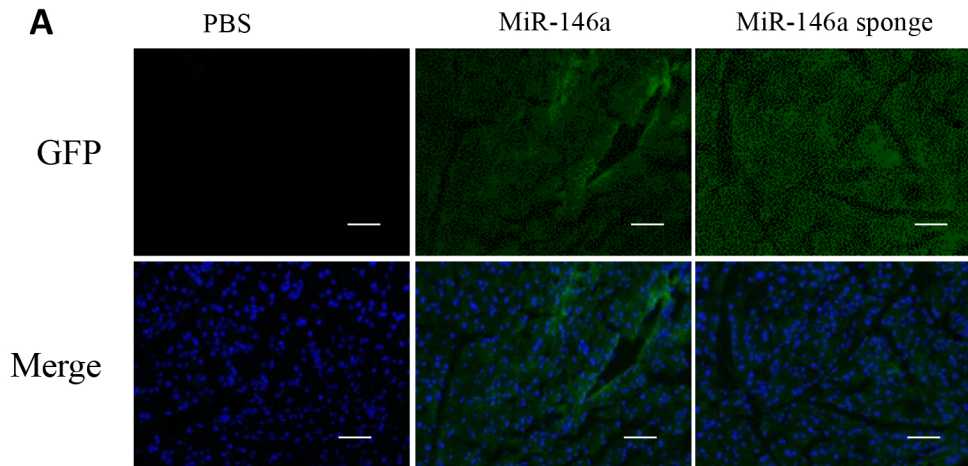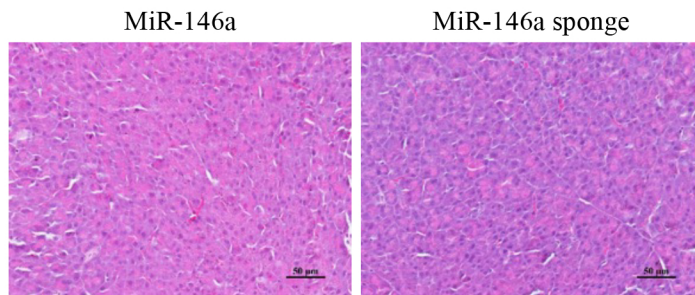

Supplement: Supplementary file 1 — Supporting information. [file IID3-13-e70163-s001.pdf]

**A**

**AAV-negative+NS**

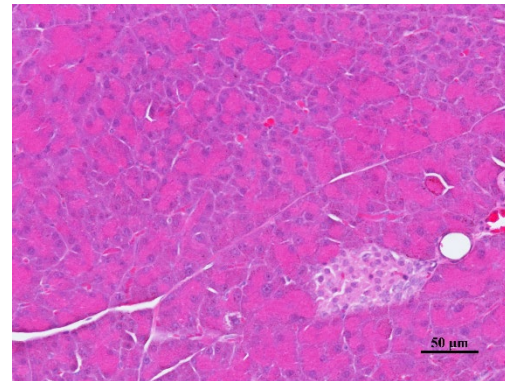

**AAV-negative+Cn**

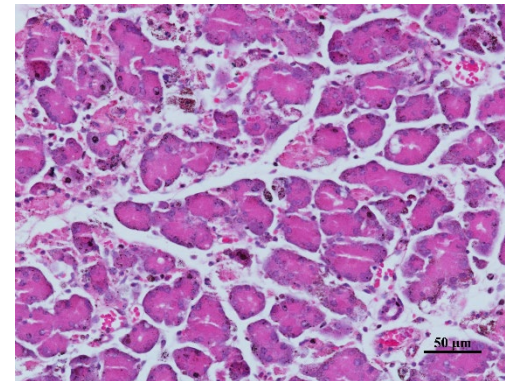

Supplement: Supplementary file 2 — Supporting information. [file IID3-13-e70163-s002.pdf]
