# Supplementary material for: MiR‐146a Reduces Inflammation in Experimental Pancreatitis via the TRAF6–NF‐κB Signaling Pathway in Mice
Source: Immun Inflamm Dis. 2025 Feb 28;13(3):e70163. doi: 10.1002/iid3.70163 (PMC11868994; doi:10.1002/iid3.70163)
Supplement: Supplementary file 3 — Supporting information. [file IID3-13-e70163-s003.docx]

**Supplement Figure Legends**

SUPPLEMENT FIGURE 1 | **The expression of GFP in pancreatic acinar cells in mice (n = 6 for each group). (A)** The expression of GFP and pancreatic histology are shown. PBS: PBS treatment. MiR-146a: miR-146a overexpression treatment. MiR-146a-sponge: miR-146a-sponge treatment.

SUPPLEMENT FIGURE 2 | **AAV-negative treatment in AP mice (n = 6 for each group). (A)** Pancreatic histology in mice. AAV-negative+NS: AAV-negative and normal saline treatment. AAV-negative+Cn: AAV-negative and caerulein treatment.
